# Study on the Regulation of Diethylene Glycol on the Hydration Process of High-Activity Calcium Oxide

**DOI:** 10.3390/ma19061132

**Published:** 2026-03-14

**Authors:** Yu Fan, Wei Guo, Yueyang Hu, Yue Zhang, Jiaqing Wang, Zhaijun Wen

**Affiliations:** 1 College of Materials Science and Engineering, Yancheng Institute of Technology, Yancheng 224002, China; fydsga@163.com (Y.F.);; 2 College of Civil Engineering, Nanjing Forestry University, Nanjing 210037, China; 3 China Building Materials Academy Co., Ltd., Beijing 100024, China

**Keywords:** reactive calcium oxide, hydration chemistry, diethylene glycol, chemical regulation, high specific surface area

## Abstract

Traditional calcium hydroxide (Ca(OH)_2_) typically exhibits low specific surface area and reactivity, significantly limiting its efficacy in industrial gas–solid reactions such as flue gas desulfurization and thermochemical energy storage. To address these limitations, this study proposes a two-stage synthesis strategy designed to enhance the surface properties and chemical activity of Ca(OH)_2_. The process involves the preparation of high-activity calcium oxide (CaO), followed by controlled hydration using diethylene glycol (DEG). Drawing on established mechanisms from cement chemistry, wherein potassium ions (K^+^) catalyze the decomposition of calcium carbonate (CaCO_3_), limestone particles (10–20 mm) were pre-soaked in a 0.1 mol/L potassium nitrate (KNO_3_) solution for 48 h prior to calcination. Characterization via X-ray diffraction (XRD), scanning electron microscopy (SEM), and Blaine Air Permeability Method analysis revealed that this pretreatment accelerated decomposition kinetics by inducing surface defects, yielding CaO with a maximum reactivity of 435.7 mL. Subsequent hydration at 80 °C with 70 wt% DEG effectively suppressed particle agglomeration and promoted the formation of thin platelet structures. The resulting Ca(OH)_2_ achieved a utilization efficiency of 98.5% and a specific surface area of 43.24 m^2^/g, demonstrating a robust technical route for fabricating high-performance calcium-based sorbents for environmental and energy applications.

## 1. Introduction

Calcium hydroxide (Ca(OH)_2_) serves as a critical inorganic precursor in diverse industrial sectors, including metallurgy, chemical synthesis, environmental remediation, and construction [1,2,3]. In flue gas desulfurization (FGD), the reactivity and specific surface area (SSA) of Ca(OH)_2_ are the primary determinants of SO_2_ removal efficiency [4,5]. However, the poor porosity and low reactivity of conventional commercial Ca(OH)_2_ often fail to meet increasingly stringent global mandates for ultra-low emission levels [6]. Furthermore, the Ca(OH)_2_ system has garnered significant interest for thermochemical energy storage (TCES) due to its high energy density, cost-effectiveness, and chemical reversibility [7,8]. Despite these advantages, Ca(OH)_2_ synthesized via traditional hydration typically exhibits low SSA and a propensity for agglomeration and sintering during cycling—constraints that substantially impede its performance in specialized applications [9,10]. Consequently, developing advanced synthesis protocols to produce highly active Ca(OH)_2_ with enhanced SSA is of profound theoretical and practical importance.

Since Ca(OH)_2_ is primarily synthesized via the hydration of calcium oxide (CaO), the physicochemical properties of the CaO precursor dictate the quality of the final product [11]. Enhancing CaO reactivity is therefore a critical initial step. While the flash calcination of limestone powder can yield high reactivity, the accumulation of fine particles often induces localized “hot spots” and non-uniform heat distribution, leading to either under-calcination or “dead-burning” [12,13]. The use of bulk limestone mitigates these issues by improving permeability and thermal gradients [14]. Furthermore, recent studies indicate that the calcination atmosphere and the presence of specific ions significantly influence decomposition kinetics [15]. For instance, Liendo et al. [16] demonstrated that exogenous ions can alter the nucleation kinetics of calcium carbonate. Drawing on cement chemistry, alkali metal ions (e.g., K^+^) are recognized as effective mineralizers that accelerate solid-state reactions by inducing lattice distortion and forming transient liquid phases [17]. Building upon these findings, a pre-immersion protocol using ionic solutions offers a dual benefit: the aqueous medium cleans the mineral surface, while dissolved ions adsorb onto the surface or penetrate the pore structure [18]. Subsequent calcination promotes surface etching and defect formation [19], thereby reducing the decomposition temperature and yielding highly reactive CaO with a refined porous microstructure [20,21].

The subsequent hydration (digestion) stage is decisive in determining the morphology and specific surface area of the final Ca(OH)_2_ product. This complex process involves the dissolution of CaO, followed by the nucleation and growth of Ca(OH)_2_ crystals [22]. While utilizing highly reactive CaO accelerates dissolution, the absence of adequate regulation often leads to significant particle agglomeration and crystal coarsening [23]. Solvent-mediated synthesis using functional additives has emerged as an effective strategy for modulating these kinetics. Specifically, organic molecules can act as crystal habit modifiers, governing nucleation and growth rates to favor high-surface-area morphologies [24]. Among various additives, alcohols and glycols have demonstrated particular efficacy; for instance, polyethylene glycol (PEG) has been successfully employed as a dispersant in CaO-based sorbent systems [25]. Notably, diethylene glycol (DEG) possesses unique chelating properties, enabling it to adsorb onto specific crystallographic planes of Ca(OH)_2_ nuclei. This adsorption provides steric hindrance, which effectively suppresses agglomeration and promotes the formation of nanostructured thin platelets [26]. Recent research further suggests that such organic additives can facilitate non-classical crystallization pathways, leading to even greater reductions in primary particle size [27].

Although significant progress has been made in calcium-based materials, there are still critical knowledge gaps in the collaborative integration of precursor activation and additive control during the hydration process. Traditional studies [28] usually treat the calcination parameters and hydration additives as independent variables, but overlook the impact of calcium oxide activity on the subsequent digestion synthesis. This study is quite different from these scattered approaches. It establishes a unified technical route that links the processing of granular limestone with the synthesis of high-specific-surface-area (SSA) Ca(OH)_2_ through high surface area (SSA) synthesis. The core innovation lies in the systematic exploration of the interaction between salt pre-impregnation and DEG-assisted dissolution—this dual regulatory strategy has largely remained unexplored. Ca(OH)_2_ with a specific surface area of 41.555 m^2^/g enables a tenfold increase in desulfurization breakthrough time compared to untreated Ca(OH)_2_ [29]. By using the surface activity of diethylene glycol (DEG) to regulate the coordination of Ca^2+^ [24] and inhibiting crystal aggregation, we synthesized Ca(OH)_2_ with a higher specific surface area of over 41.555 m^2^/g, and the desulfurization performance of higher-specific-surface-area calcium hydroxide will be better. In addition to providing a reliable synthesis method, this study also provides a theoretical basis, challenges the traditional “single-stage optimization” model, and greatly promotes the design and development of high-performance materials for desulfurization, denitrification, and energy storage.

## 2. Experimental Methods

### 2.1. Raw Materials and Reagents

**Figure 1:**
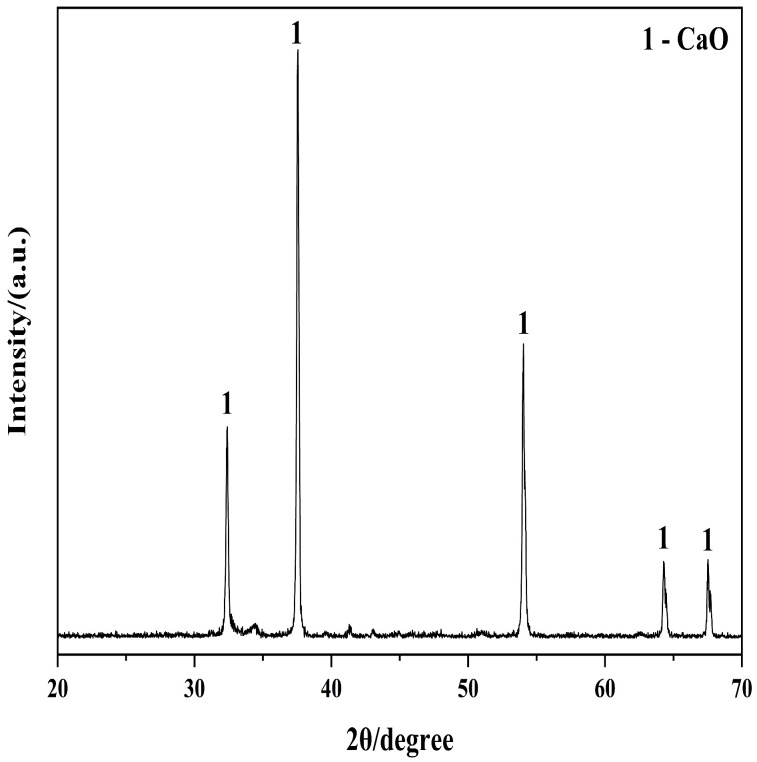
X-ray Diffraction Pattern of Raw Limestone.

Natural limestone (the composition of which is detailed in Table 1 and Figure 1), potassium nitrate (KNO_3_, AR, Sinopharm Chemical Reagent Co., Ltd., Shanghai, China), magnesium sulfate (MgSO_4_, AR, Sinopharm Chemical Reagent Co., Ltd., Shanghai, China), sodium carbonate (Na_2_CO_3_, AR, Sinopharm Chemical Reagent Co., Ltd., Shanghai, China), and magnesium nitrate heptahydrate (Mg(NO_3_)_2_·7H_2_O, AR, Sinopharm Chemical Reagent Co., Ltd., Shanghai, China) were the primary raw materials used in this study. Diethylene glycol (DEG, C_4_H_10_O_3_, purity > 99%, Sinopharm Chemical Reagent Co., Ltd., Shanghai, China) served as a functional additive to modify the hydration process. Deionized water was used to prepare all solutions and conduct the hydration experiments.

### 2.2. Preparation and Reactivity Assessment of Active CaO

Bulk limestone was crushed and sieved to obtain a particle size fraction between 10 and 20 mm. The resulting granules were soaked in 0.1 mol/L aqueous solutions of various salts (KNO_3_, MgSO_4_, Na_2_CO_3_, and Mg(NO_3_)_2_·7H_2_O) for 48 h. Calcination of the pre-soaked limestone was performed in a muffle furnace under the following conditions: samples were heated to 800 °C at a rate of 15 °C/min and held for 1 h, then further heated to 1050 °C and maintained for 3 h. Upon completion of the calcination process, samples were rapidly cooled to room temperature in a desiccator.

Prior to characterization and hydration, the resulting CaO particles were milled and sieved to pass through a 150 μm mesh. This ensured uniformity and prevented particle size variations from influencing the reactivity tests and XRD analysis.

The reactivity of the produced CaO was assessed using a standardized titration technique. Initially, 50.0 g of 1–5 mm CaO grains were sieved and stored in a desiccator. Subsequently, 2000 mL of distilled water (maintained slightly above 40 °C) and 8–10 drops of 5 g/L phenolphthalein solution were prepared. Under constant stirring, the CaO sample was added to the solution in a single addition. Simultaneously, a 4 mol/L hydrochloric acid (HCl) solution was titrated into the mixture. The procedure was concluded after exactly 10 min, and the volume of HCl consumed at this interval was recorded as the measure of CaO reactivity.

### 2.3. Synthesis of High-Specific-Surface-Area Ca(OH)_2_

The hydration process of active calcium oxide (CaO) is carried out in a constant temperature water bath, with the water temperature maintained at a constant level to ensure a stable reaction environment. Deionized water is mixed with diethylene glycol (DEG) at five different mass concentrations, namely 50%, 60%, 70%, 80%, and 90%. Then, the deionized water–DEG mixed solvent is mixed with the active CaO, with a liquid–solid ratio of 4:1 (volume/mass, that is, 4 mL of the mixed solvent per 1 g of active CaO, based on the initial dry mass of CaO to ensure consistent solid loading in all experimental groups). The mixture is uniformly stirred with a stirrer at a constant speed of 300 revolutions per minute for 15 min to promote sufficient contact between CaO and the mixed solvent and ensure a uniform hydration process. To terminate the hydration process, anhydrous ethanol is added to the reaction mixture, with the addition volume ratio to the mixed solvent (that is, the deionized water–DEG mixture) being 1:1 (that is, 1 m of the deionized water–DEG mixture is added with 1 m of anhydrous ethanol). Two synergistic physical and chemical mechanisms are used to terminate the hydration process: firstly, ethanol undergoes rapid solvent exchange with the mixed solvent, replacing free water and weakly bound water on the surface and pores of calcium oxide particles, which significantly reduces the water activity in the system and deprives the hydration reaction of the necessary medium; secondly, ethanol acts as a reaction inhibitor, reducing the dissolution rate of calcium ions in calcium oxide and inhibiting the diffusion of ions in the system, thereby slowing down or even preventing the formation and growth of calcium hydroxide (Ca(OH)_2_) hydration products from a kinetic perspective. After terminating the reaction, the resulting mixture is transferred to a drying oven and dried at a constant temperature of 105 °C for 6 to 8 h (until the mass remains stable), to completely remove residual solvents and water.

### 2.4. Characterization Techniques

The crystalline phases were determined based on an observation of an X-ray diffractometer (DX-2700, Dandong Fangyuan Instrument Co., Ltd., Dandong, China) with Cu Kα radiation (λ = 1.5406 Å) was applied at 35 kV and 30 mA. The scans were conducted between the 2θ values of 5–80° by 0.02° step size. Quantitative phase analysis was carried out in HighScore Plus through Rietveld refinement.

Analysis of Particle Size (PSA): Measured with Laser Diffraction Particle Size Analyser (LS13320, Beckman Coulter, Inc., Brea, CA, USA). The diffraction optical model used to calculate particle size distribution was Fraunhofer. In order to inhibit the occurrence of secondary hydration in measuring process, the dispersing agent used was ethanol.

Morphology: The FE-SEM (Field Emission Scanning Electron Microscopy) (FEI Company, Hillsboro, OR, USA) was used to examine the microscopic morphology. To improve its conductance, a sample was first coated with gold before being imaged.

Specific Surface Area: Specific Surface Area (SSA) was determined with FBT 9 Analyzer(Leiyun, Shanghai, China). Several measurements were done on the samples and the mean values were used to minimize errors.

Heat Treatment and Organic Component: In this study, thermogravimetric analysis (TGA) and differential thermal analysis (DTA) were performed with a multi-purpose thermal analyzer. The samples were heated starting at room temperature and reaching temperatures of 700 °C with a heating rate of 10 °C/min under a nitrogen atmosphere. The DEG loading on Ca(OH)_2_ particles was estimated through determining the weight loss within the temperature interval between 200 and 400 °C, which is related to the organic additive decomposition.

## 3. Results and Discussion

### 3.1. Limestone Decomposition & Active CaO

#### 3.1.1. Analysis of Limestone Decomposition Products

**Figure 2:**
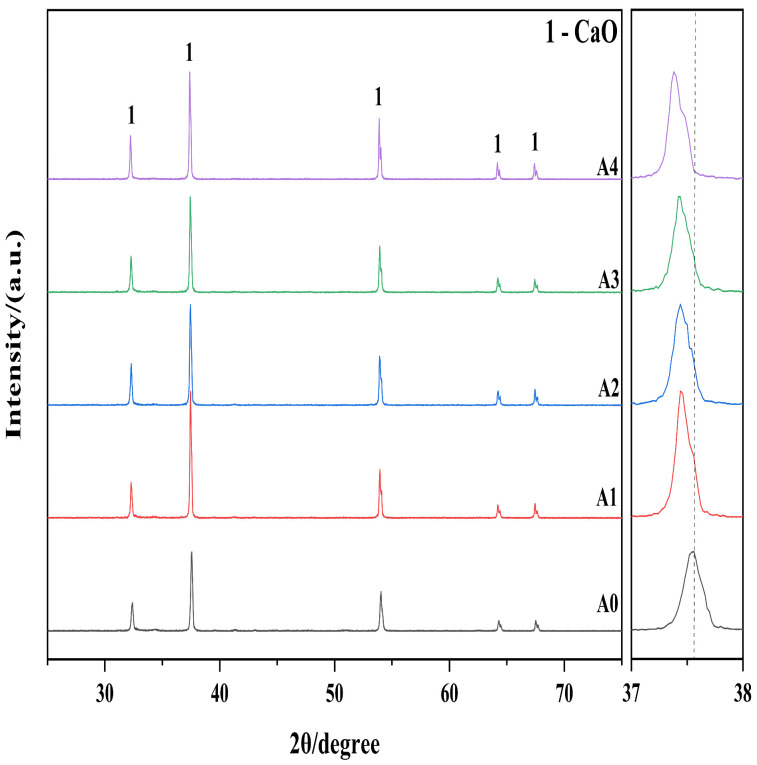
XRD patterns of active CaO prepared from pre-immersed limestone.

The limestone blocks were immersed in four different salt solutions, namely, KNO_3_, MgSO_4_, Na_2_CO_3_ and Mg(NO_3_)_2_·7H_2_O, with a period of 48 h which would be identified as specimens A1, A2, A3 and A4, respectively. Without pre-soaked limestone acted as the control group and was labeled as specimen A0. The obtained active products of CaO were analyzed by X-ray diffraction and the results are given in Figure 2.

Figure 2 illustrates the X-ray diffraction (XRD) patterns of the active CaO samples (A0–A4). Notably, only CaO diffraction peaks were observed; no characteristic peaks for impurities, such as Ca(OH)_2_ (typically appearing at approximately 18° or 34.1°), were detected. This confirms that the synthesis and preservation protocols successfully prevented moisture ingress, thereby maintaining high phase purity. To clarify the structural changes induced by the modification, a magnification of the characteristic (200) peak is provided on the right side of Figure 2. A shift toward lower diffraction angles is observed from sample A0 to A4. According to Bragg’s Law (2dsinθ= nλ), this shift indicates an increase in interplanar spacing (d-spacing). This lattice expansion provides direct structural evidence of a “mineralizing effect”, analogous to mechanisms established in cement chemistry. As demonstrated in clinkerization studies [30], the incorporation of alkali metals, such as K^+^, into the calcium-rich lattice induces structural distortion and reduces the eutectic temperature. This phenomenon, termed the fluxing effect, facilitates mass transfer and creates lattice defects.

Furthermore, it was found that the lattice expansion of sample is believed to be caused by the addition of K^+^ ions with the ionic radius of 1.38 Å, which is much bigger when compared to the ionic radius of Ca^2+^ ions (1.00 Å), and this causes local deformation of the host lattice, confirming the defect engineering mechanism. Moreover, the widening of the diffraction peaks points to an enhanced lattice strain.

Quantitative analysis derived using the Williamson–Hall approach [31].

(1) $$\beta_{hkl}\cos\theta =\frac{K\lambda }{D}+4\varepsilon \sin\theta$$

where *β_hkl_* is the half-width, *θ* is the diffraction angle, *ε* represents the lattice strain, and D is the crystallite size. The calculated results are listed in Table 2

Table 2 lists the structural parameters obtained through the Williamson–Hall analysis. Quantitative findings are in perfect correlation with qualitative assessments of the shift in the XRD peaks. To be more precise, the lattice parameter value of the unmodified sample (A0) is 4.787 Å, but it reaches its highest point of 4.805 Å at sample A1. This quantifiable expansion quantitatively demonstrates that the bigger K^+^ ions have indeed been introduced into the CaO crystal lattice, which in turn has increased the size of the unit cell.

Furthermore, the structural modification resulted in a distinct upward trend in microstrain (*ε*). The untreated sample (A0) exhibited the lowest microstrain at 0.93 × 10^−3^, whereas the modified samples displayed higher values, reaching a maximum of 1.24 × 10^−3^ for sample A1. This significant increase in lattice strain suggests that K^+^ ion incorporation generates substantial point defects and localized structural distortions within the host lattice. Concurrently, the crystallite size increased from 64.6 nm (A0) to a range of 78.4–121.2 nm for the modified samples. During calcination, KNO_3_ likely forms a transient liquid phase at the grain boundaries, accelerating mass transfer and promoting grain boundary diffusion, a mechanism analogous to liquid-phase sintering in cement clinker production. This implies that the thermal calcination process, facilitated by the availability of alkali metal ions, enhances grain boundary diffusion and promotes crystallite growth. These structural alterations—specifically lattice expansion, increased microstrain, and shifts in crystallite size—are expected to enhance the reactivity of the active CaO by increasing the density of active sites and lattice defects.

The activity of all calcium oxide samples was then tested, and the results are shown in Table 3.

The activity of the calcium oxide samples was determined by the standard titration method. The average activity values ranged from 357.1 mL (A0, control group) to 435.7 mL (A1), indicating a significant increase in reaction activity after specific treatment. Sample A1 had the highest activity, with a standard deviation of 5.19 mL, showing extremely high reproducibility. The order of sample activity was A1 > A4 > A3 > A2 > A0, suggesting that the preparation conditions of A1 were more effective in promoting the active sites of calcium oxide, that is, the calcium oxide sample obtained after pre-soaking with KNO_3_ and then calcination was better.

#### 3.1.2. Differential Thermal Analysis

**Figure 3:**
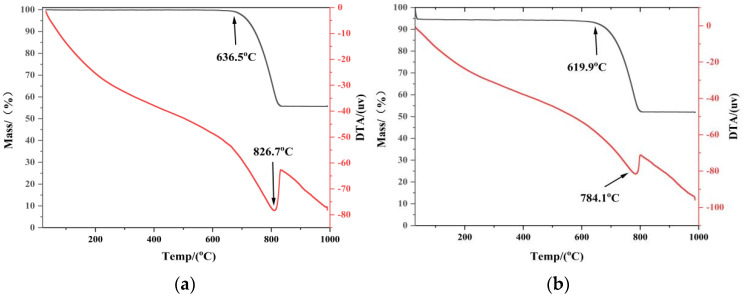
TG-DTA profiles of limestone specimens (**a**) untreated sample A0, and (**b**) KNO_3_-treated sample A1.

In order to clarify more the catalytic action of KNO_3_ on limestone breakdown, the differential thermal analysis was done on specimens A0 and A1 with the findings as illustrated in Figure 3.

The untreated limestone (A0) begins to lose mass at a threshold temperature of 636.5 °C, with an endothermic reaction corresponding to the decomposition of CaCO_3_, as illustrated in Figure 3a. A sharp endothermic peak, representing the maximum decomposition rate, is observed at 826.7 °C. In contrast, the KNO_3_-treated sample (A1) exhibits a significant shift toward lower temperatures (Figure 3b). Decomposition initiates at 619.9 °C, with the peak recorded at 784.1 °C. Notably, the peak decomposition temperature of the treated specimen is 42.6 °C lower than that of the untreated sample. This substantial decrease confirms that KNO_3_ pre-soaking effectively accelerates the rate of CaCO_3_ decomposition. The observed reduction in decomposition temperature is attributed to the “flux effect” of potassium salts. Similar to the role of mineralizers in cement sintering, the presence of alkali metals creates a localized low-melting-point environment that lowers the energy barrier for the solid-state reaction [15]. Reducing the decomposition temperature is beneficial for the final product, as high calcination temperatures typically lead to CaO grain coarsening and a reduction in specific surface area. Consequently, lower thermal consumption not only improves energy efficiency but also potentially enhances the reactivity of the resulting CaO.

Moreover, the results of the thermal analysis also confirm the outcomes of the XRD analysis. It is believed that the incorporation of K^+^ ions leads to the distortion of the lattice, which causes the reduction in the decomposition temperature. This lattice strain makes CaCO_3_ structure unstable causing a decrease in the energy barrier that can be overcome through decomposition.

#### 3.1.3. Microscopic Morphology Analysis

**Figure 4:**
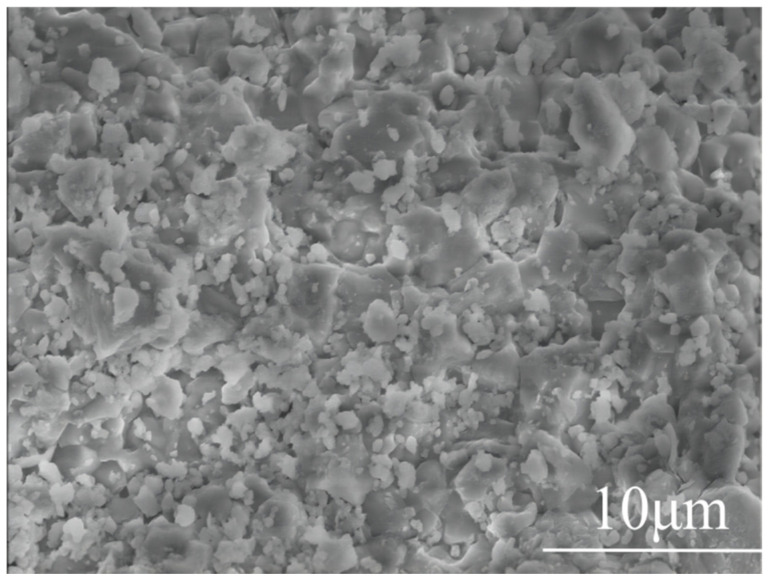
SEM micrograph showing the surface morphology of the KNO_3_-pretreated limestone.

Figure 4 illustrates the microscopic surface morphology of the limestone following pretreatment via immersion in KNO_3_. It is evident that the treated limestone surface exhibits a high degree of roughness, characterized by a distribution of small pores and micro-cracks. During the immersion stage, the aqueous salt solution effectively penetrated the capillary channels of the rock matrix. Subsequently, during drying and the initial heating phases, the rapid evaporation of water, combined with the catalytic influence of K^+^ ions, promoted the expansion of these pores and the formation of additional fractures. This microstructural evolution is significant as it enhances the mass and heat transfer properties of the particles, thereby increasing the reactivity of the limestone during the subsequent decomposition of CaCO_3_.

**Figure 5:**
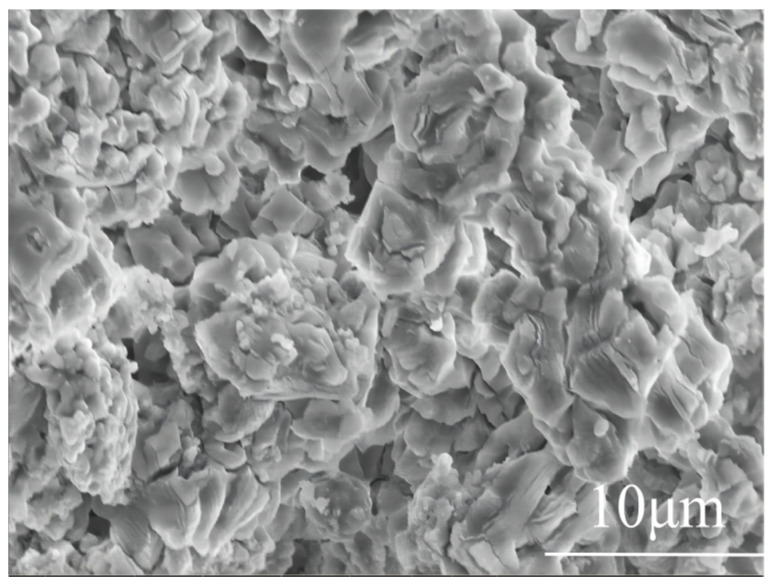
SEM micrograph of the active CaO obtained from the calcination of KNO_3_-treated limestone.

Figure 5 presents the SEM imagery of the CaO product obtained from the calcination of the KNO_3_-modified limestone. The resulting CaO crystals primarily form granular, irregular aggregates with crystallite sizes ranging from approximately 0.1 to 5 μm. These crystals exhibit a porous, loosely packed arrangement rather than a compact structure, maintaining distinct intergranular spacing. This morphological evolution is facilitated by the formation of a transient liquid phase induced by the potassium nitrate, which accelerates mass transfer and leaves behind a porous framework upon decomposition. Furthermore, the incorporation of K^+^ ions into the interstices or lattice vacancies of the CaCO_3_ crystal structure induces substantial lattice distortion and the formation of numerous crystallographic defects. These defects alter the crystal growth kinetics, inhibiting over-sintering and ultimately yielding the observed refined geometry. These morphological features ensure that the final CaO possesses an exceptionally high specific surface area, thereby increasing the density of active sites and overall surface reactivity.

### 3.2. Preparation of Ca(OH)_2_ via Solvent-Mediated Hydration

**Figure 6:**
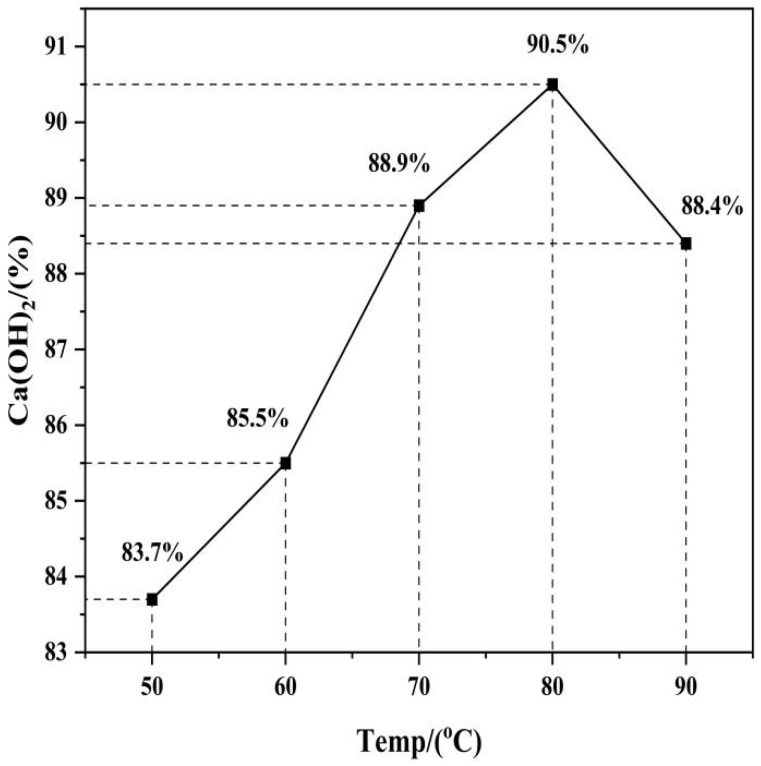
Conversion Yield of Ca(OH)_2_ at Various Water Bath Temperatures.

Before starting the hydration process, it is crucial to set the optimal temperature of hydration. Therefore, an organized research was conducted to explore the effect of hydration temperature (50, 60, 70, 80, and 90 °C) on the conversion yield of Ca(OH)_2_. The deionized water–DEG mixed solvent was mixed with the active calcium oxide, with a liquid–solid ratio of 4:1. The mixture was homogenously stirred at a constant speed of 300 revolutions per minute using a stirrer for 15 min to ensure adequate contact between the calcium oxide and the mixed solvent and to ensure a uniform hydration process. The yield of Ca(OH)_2_ was determined using quantitative XRD analysis, and the result was presented graphically in Figure 6.

The conversion of Ca(OH)_2_ is depicted in Figure 6 and indicates the consistent increase with the increase in temperature that peaks with the highest conversion yield as high as 90.5% at 80 °C. An additional elevation in the temperature to 90 °C leads to a decrease in conversion efficiency (88.4%). Therefore, the appropriate temperature used in the next series of the hydration experiments is set to be 80 °C.

The hydration reaction of CaO with water follows this stoichiometric relationship:

(2) $$\mathrm{CaO}(s)+H_{2}O(l)={\mathrm{Ca}(\mathrm{OH})}_{2}(s)+64.9\;\mathrm{kJ}$$

According to theory, one mole of CaO (56.08 g/mol) consumes one mole of water (18.02 g/mol) in the process of stoichiometric conversion. Due to the great amount of heat produced during the process (64.9 kJ), however, some of the water can be partially vaporized, which will result in incomplete hydration caused by dehydration. It means that the water needs to be supplied over the theoretical stoichiometric demand. In the present experiments, an excess of deionized water of 20–30% by moles was used to obtain full reaction.

In the case of the solvent-mediated hydration, different combinations of diethylene glycol (DEG) and deionized water were the medium of hydration. Table 4 describes the compositions of the particular solvent systems in detail.

#### 3.2.1. Influence of Diethylene Glycol Content on Calcium Hydroxide Particle Size Distribution

**Figure 7:**
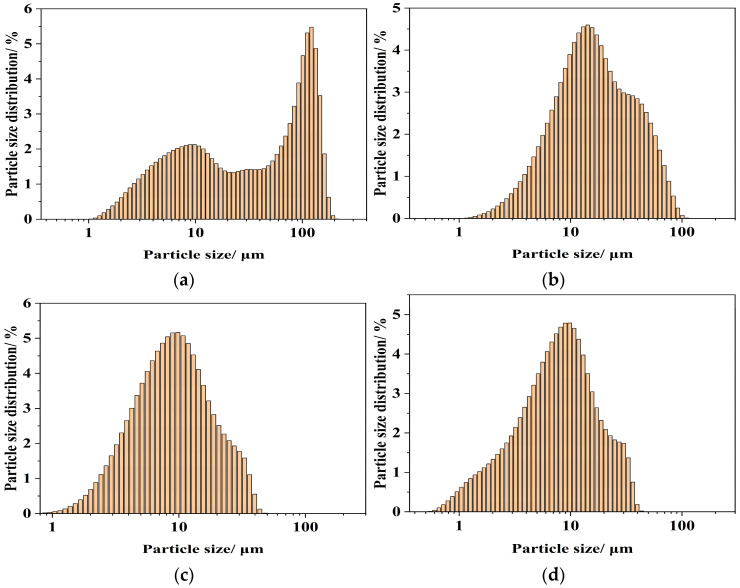

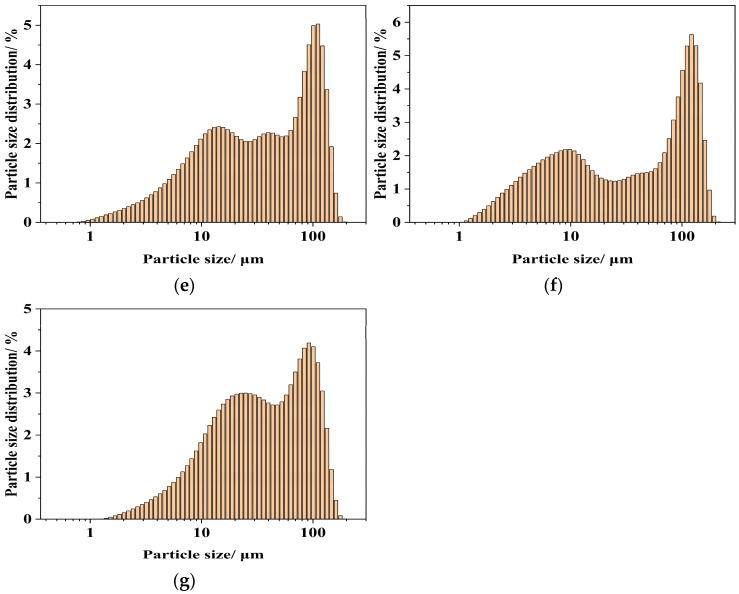
Particle Size Distribution of CaO Precursor and Ca(OH)_2_ Prepared with Varying DEG Content: (**a**) CaO, (**b**) 50 wt% DEG, (**c**) 60 wt% DEG, (**d**) 70 wt% DEG, (**e**) 80 wt% DEG, (**f**) 90 wt% DEG, and (**g**) 100 wt% H_2_O.

The particle size distributions (PSD) of the raw CaO precursor and the Ca(OH)_2_ products synthesized with varying DEG concentrations are presented in Figure 7.

As shown in Figure 7a, the milled raw CaO precursor exhibits a typical bimodal particle size distribution with a D_90_ of 136.40 μm, indicating a wide size range resulting from mechanical milling. This bimodal distribution is the macroscopic manifestation of structural defects (including lattice distortion, surface microcracks, and grain boundary defects) introduced by mechanical milling, which is the key reason for the high hydration activity of the active CaO used in this study. The uneven distribution of these defects leads to the differential hydration reactivity of CaO surface, as verified by the residual unreacted CaO in the pure water hydration system (Table 5). A similar bimodal profile with a D_90_ of 112.9 µm is observed when the dehydration reaction is conducted without DEG (Figure 7g, 100% H_2_O). This marginal decrease in particle size suggests that water alone is insufficient to disrupt the aggregates formed during the reaction, leading to persistent agglomeration.

In contrast, the inclusion of DEG dramatically alters the particle size distribution. As the DEG concentration increases from 50 wt% to 70 wt% (Figure 7b–d), the PSD shifts to a unimodal and narrower distribution. Specifically, the D_90_ values decrease significantly to 50.50 μm (50 wt% DEG), 24.15 μm (60 wt% DEG), and 22.33 μm (70 wt% DEG). These values represent a 60–80% reduction compared to the DEG-free control. This trend suggests that DEG acts as an effective dispersant within this concentration range, inhibiting particle agglomeration through steric hindrance and modulating nucleation–growth kinetics.

However, further increasing the DEG concentration results in a reversal of this trend. Broadened distributions with higher D_90_ values of 123.6 μm and 141.4 μm are observed for samples prepared with 80 wt% and 90 wt% DEG, respectively (Figure 7e,f). This phenomenon can be attributed to the physicochemical properties of the solvent system. High DEG concentrations significantly increase the viscosity of the reaction medium, thereby hindering mass transfer and the diffusion of water molecules to the CaO surface. Consequently, the local supersaturation required for the rapid formation of nuclei is disrupted; under these highly viscous conditions, nuclei aggregation into large clusters is favored over dispersion.

**Figure 8:**
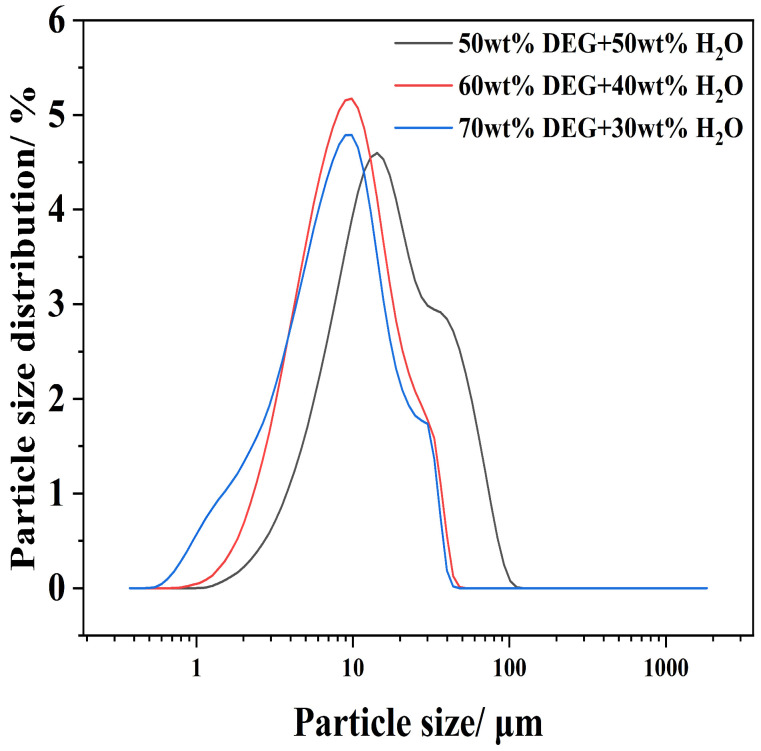
Particle Size Distribution of Ca(OH)_2_ Prepared with Varying DEG Content.

On the basis of these findings, it was determined that the synthesized samples containing 50–70 wt% DEG exhibited a very fine particle size. The comparisons between their PSD curves are given in Figure 8.

Figure 8 demonstrates that the 70 wt% DEG concentration produces the most uniform and finest particles, resulting in the highest specific surface area within the series. Consequently, 70 wt% DEG is identified as the optimal solvent concentration required to minimize particle size and maximize dispersion within this system.

#### 3.2.2. X-Ray Diffraction (XRD) Analysis of Ca(OH)_2_ Synthesized with Varying DEG Content

**Figure 9:**
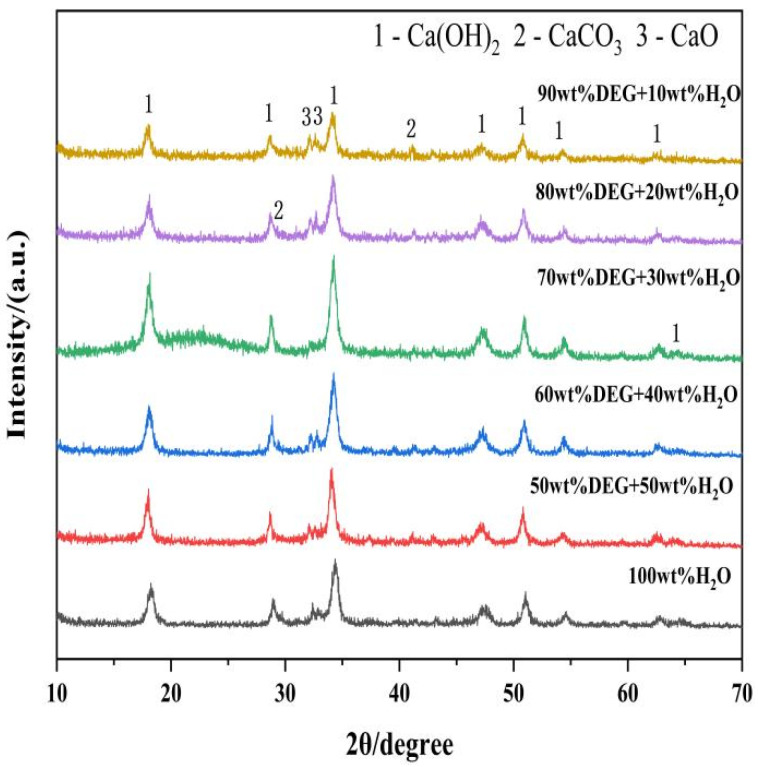
X-ray Diffraction Patterns of Ca(OH)_2_ Synthesized with Varying DEG Content.

The X-ray diffraction (XRD) patterns of the Ca(OH)_2_ products synthesized with varying concentrations of diethylene glycol (DEG) are presented in Figure 9. The diffraction peaks were indexed to portlandite (Ca(OH)_2_), calcite (CaCO_3_), and unreacted lime (CaO). To quantitatively assess the hydration efficiency, Rietveld refinement analysis was performed, and the results are summarized in Table 5.

Quantitative analysis of the control sample hydrated in deionized water (0 wt% DEG) revealed a Ca(OH)_2_ yield of 90.5%, with a significant amount of unreacted CaO (8.4%) remaining. This suggests that hydration in pure water is kinetically constrained, likely due to the formation of a dense passivation layer on the CaO surface that inhibits water diffusion.

Conversely, the presence of DEG promotes the hydration reaction. In the concentration range of 50–70 wt% DEG, the residual CaO concentration decreases as the DEG content increases. At 70 wt% DEG, the Ca(OH)_2_ yield reaches its maximum value of 98.5%. This improvement indicates that DEG acts not only as a solvent component but also as a crystal growth regulator. The hydroxyl groups in DEG likely chelate with Ca^2+^ ions, thereby modulating their reactivity and altering nucleation kinetics. Furthermore, the adsorption of DEG molecules onto the nascent Ca(OH)_2_ nuclei provides steric hindrance, which prevents particle aggregation and maintains the exposure of fresh surface area for continued reaction.

However, this promoting effect is concentration-dependent. At higher DEG concentrations (80 wt% and 90 wt%), the Ca(OH)_2_ yield declines to 92.4% and 92.1%, respectively, with unreacted CaO levels rising to 6.2–6.6%. This reversal is attributed to the physicochemical properties of the reaction medium. In excess DEG, the solution viscosity increases significantly, which hinders the mass transfer of water molecules to the CaO surface and subsequently reduces the hydration rate, despite the presence of the additive. It is worth noting that DEG can effectively improve the utilization efficiency of CaO structural defects: at the optimal 70 wt% DEG concentration, the residual CaO is reduced to 0% (Table 5), which is because the chelation of DEG with Ca^2+^ at the CaO defect sites delays the formation of passivation layer, allowing water molecules to fully contact the internal defect sites of CaO particles and realize complete hydration.

**Figure 10:**
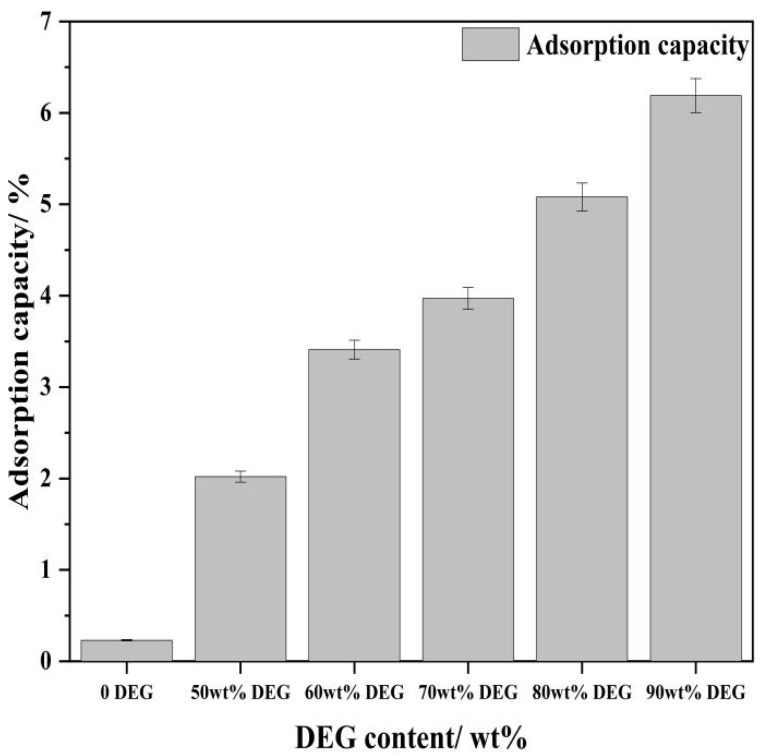
Adsorption capacity of DEG on Ca(OH)_2_ particles synthesized with varying solvent compositions.

To further elucidate the interaction between DEG and the calcium hydroxide particles, the adsorption capacity of DEG on the product surfaces was determined (Figure 10). The sample synthesized without DEG exhibited negligible organic adsorption (0.23%), which may be attributed to background noise or minor moisture absorption. In contrast, samples prepared with DEG showed a monotonic increase in adsorption capacity, rising from 2.02% at 50 wt% DEG to 6.19% at 90 wt% DEG.

As illustrated in Figure 10, at a diethylene glycol (DEG) mass fraction of 70 wt%, the adsorption amount remains below 4%, which facilitates effective particle dispersion and morphological control. In contrast, the high adsorption values (5.08–6.19%) observed at elevated DEG concentrations (80–90 wt%) are closely linked to the decreased hydration rate. Under these high-concentration conditions, a dense, multilayer film of adsorbed DEG molecules may form at the CaO/Ca(OH)_2_ interface. This layer acts as a physical barrier that prevents water molecules from reaching the particle surface, thereby inhibiting the subsequent hydration reaction. Consequently, by balancing the surface modification effects with the reaction kinetics, 70 wt% is identified as the optimal DEG concentration.

Furthermore, the chelation between DEG hydroxyl groups and Ca^2+^ is further supported by the direct DEG adsorption capacity data (Figure 10) in this study—DEG exhibits a significant and concentration-dependent adsorption on Ca(OH)_2_ surfaces, which is the direct physical evidence of the specific interaction between DEG and Ca^2+^ on the crystal surface. This chelation not only modulates Ca^2+^ reactivity but also forms a steric hindrance on the CaO/Ca(OH)_2_ interface, avoiding the rapid formation of a dense passivation layer and thus promoting the continuous hydration reaction.

#### 3.2.3. Influence of DEG Content on the Specific Surface Area of Ca(OH)_2_

**Figure 11:**
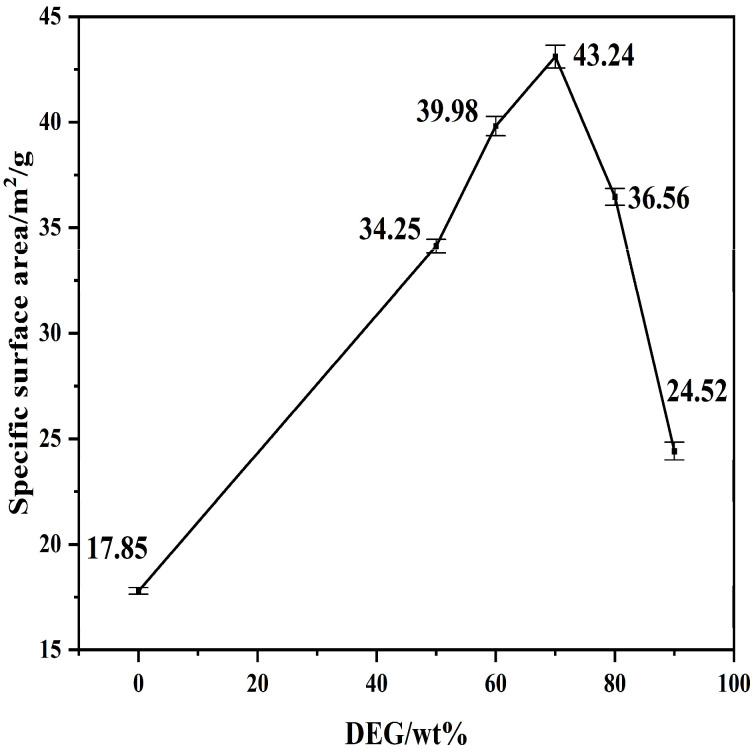
Specific Surface Area of Ca(OH)_2_ Synthesized with Varying DEG Content.

Figure 11 shows the specific surface area (SSA) of the Ca(OH)_2_ products produced at various DEG mass fractions.

The specific surface area (SSA) of the control sample prepared with deionized water (0 wt% DEG) is relatively low (17.85 m^2^/g). The addition of DEG induces a remarkable increase in SSA, reaching 34.25 m/g^2^ at 50 wt% and peaking at 43.24 m/g^2^ at 70 wt% DEG. This represents a substantial improvement of approximately 142% compared to the DEG-free baseline. This significant enhancement aligns with the particle size measurements, confirming that DEG acts as an effective surfactant at these concentrations. By adsorbing onto crystal surfaces, DEG provides a steric barrier that inhibits particle agglomeration and facilitates the development of smaller, more porous crystallites with higher surface-to-volume ratios.

However, the trend reverses sharply beyond the optimal threshold of 70 wt%. The SSA values decrease to 36.56 m^2^/g at 80 wt% and further decline to 24.52 m^2^/g at 90 wt% DEG. This reduction is consistent with the particle coarsening and the broadening of particle size distributions observed in the PSD analysis (Figure 7). This behavior is attributed to the high viscosity of the reaction medium at elevated DEG levels, which restricts mass transfer and ionic mobility. Such conditions likely inhibit the nucleation rate and promote the coalescence of nuclei into larger, denser aggregates, thereby reducing the total accessible surface area. Consequently, 70 wt% DEG is identified as the optimal concentration for maximizing the specific surface area of the Ca(OH)_2_ product.

#### 3.2.4. Influence of DEG Content on the Microstructure of Ca(OH)_2_

**Figure 12:**
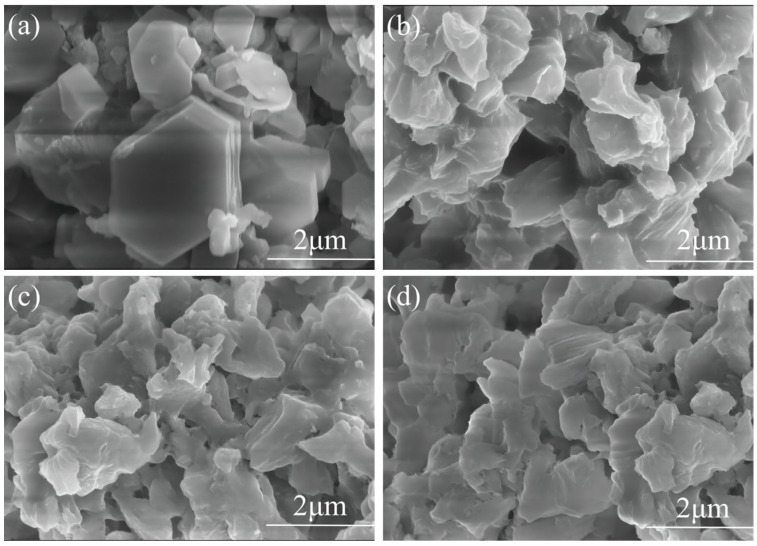
Influence of DEG Concentration on the Resulting Morphological Modifications of Synthesized Ca(OH)_2_: (**a**) 100 wt%H_2_O, (**b**) 50 wt% DEG, (**c**) 60 wt% DEG, and (**d**) 70 wt% DEG.

Figure 12 presents SEM images of Ca(OH)_2_ prepared with varying concentrations of DEG, clearly depicting the morphological evolution induced by the organic additive.

In the control sample synthesized in pure water (Figure 12a), the Ca(OH)_2_ crystals exhibit the standard hexagonal platelet morphology characteristic of portlandite. These crystals are well-crystallized with smooth surfaces and well-defined edges, typically ranging from 2 to 4 μm in size. The thickness of these platelets indicates that crystal growth along the c-axis was not significantly inhibited in the aqueous phase.

The introduction of DEG induces a progressive shift in the crystal habit. At 50 wt% and 60 wt% DEG (Figure 12b,c), the regular hexagonal geometry is disrupted. The crystals exhibit a non-homogeneous, disordered arrangement with irregular and poorly defined boundaries. Although the particle size is reduced compared to the control, the crystallites still exhibit some degree of aggregation, resulting in dense clusters of irregular flakes.

The optimal concentration of 70 wt% DEG results in a radical structural transformation (Figure 12d). The microstructure evolves into an open, porous network composed of ultrathin, crumpled nanosheets. In contrast to the large platelets in Figure 12a, these lamellae are exceptionally thin—appearing semi-transparent at the edges—and are randomly oriented, creating significant void spaces between particles. This “flower-like” or flaked architecture inhibits the dense packing of layers, thereby maximizing the accessible surface area. This observation is perfectly consistent with the BET results, which identified the 70 wt% DEG sample as having the highest specific surface area (43.24 m^2^/g).This specific surface area is significantly superior to that of most calcium hydroxide(Appendix A).

**Figure 13:**
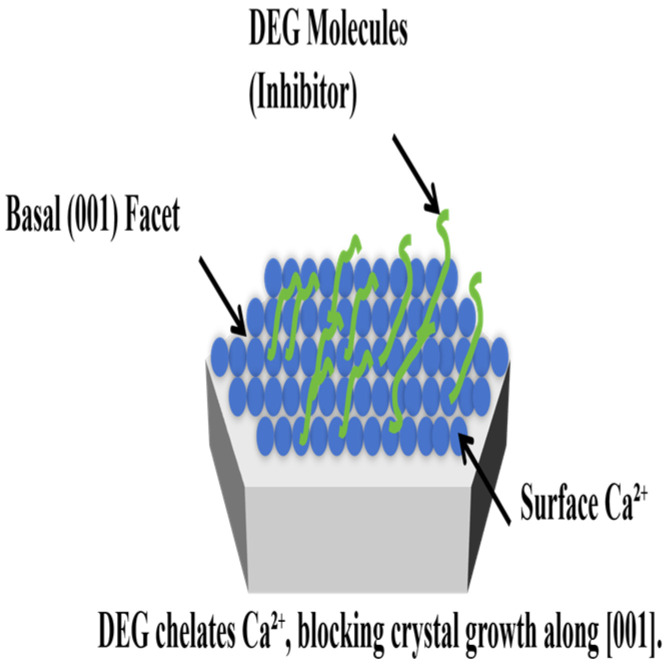
Schematic illustration of the proposed growth mechanism for Ca(OH)_2_ crystals.

Mechanism of Morphological Modification: This significant refinement is directly supported by the DEG adsorption capacity (Figure 10) and particle size distribution (Figure 7) data in this study, and is attributed to the selective adsorption and chelation of DEG molecules with Ca^2+^. DEG inhibits crystal growth by coordinating (chelating) with Ca^2+^ species on the nuclei surfaces, preferentially adsorbing onto the (001) basal planes of the Ca(OH)_2_ crystals. This adsorption creates a steric barrier that effectively blocks growth along the stacking direction (c-axis), forcing the crystals to develop into two-dimensional nanosheets rather than thick platelets (Figure 13). At the optimal 70 wt% DEG concentration, the moderate adsorption amount (<4%) balances the steric hindrance effect and hydration reaction kinetics, avoiding both insufficient dispersion and excessive interface blocking. Additionally, the appropriately increased viscosity of the solvent due to DEG reduces the collision frequency of the nuclei, preventing the formation of dense aggregates and preserving a loose, high-surface-area morphology.

#### 3.2.5. Thermogravimetric–Differential Thermal Analysis (TG-DTA) of Ca(OH)_2_ as a Function of DEG Content

**Figure 14:**
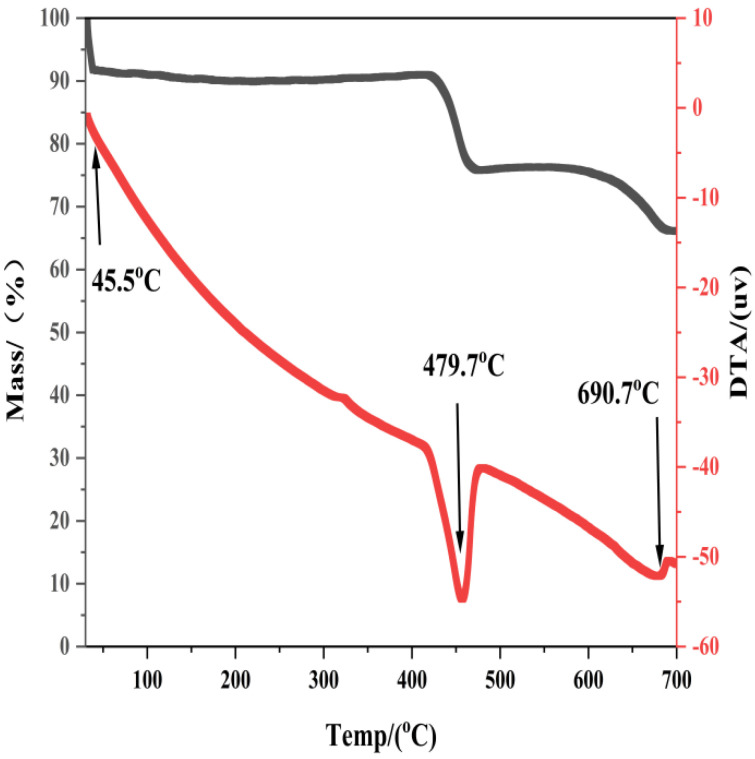
Thermogravimetric–Differential Thermal Analysis (TG-DTA) of Ca(OH)_2_ Synthesized with 70 wt% DEG.

The TG-DTA profiles of the synthesized Ca(OH)_2_ (70 wt% DEG) are shown in Figure 14. Three important physicochemical properties are identified through thermal analysis, which is related to the purity of the material, its metastability, and the reactivity of its surface.

To begin with, the analysis confirms the absence of residual organic matter. Although the boiling point of DEG is approximately 245 °C, the TG curve exhibits a stable plateau with negligible mass loss between 200 and 400 °C. This indicates that although DEG functions as an effective morphological directing agent during the liquid-phase synthesis process, due to the relatively small amount of its adsorption, differential thermal analysis shows almost no change, or it may have been removed during the process, leaving no organic residue on the Ca(OH)_2_ particles.

Second, this significant downward shift in the decomposition temperature (ΔT ≈ 30–100 °C) was directly correlated with the high specific surface area (SSA = 43.24 m^2^/g) of the DEG-modified Ca(OH)_2_. For the pure water-hydrated Ca(OH)_2_ with a low SSA of 17.85 m^2^/g, the decomposition temperature was around 510 °C (consistent with the literature [29]. In contrast, the 70 wt% DEG-modified sample with the highest SSA (43.24 m^2^/g) exhibited the most pronounced temperature shift, which was attributed to the nanostructure-induced surface energy enhancement of Ca(OH)_2_. According to the Kelvin equation and relevant studies [32], the increased SSA of Ca(OH)_2_ leads to a higher surface-to-volume ratio, resulting in the destabilization of surface hydroxyl groups (-OH) and a reduction in the activation energy required for dehydroxylation. This phenomenon confirmed that the temperature shift in DTA curves could serve as a reliable indirect indicator of the nanostructure of Ca(OH)_2_, with a higher SSA corresponding to a more significant downward shift in decomposition temperature. Furthermore, the results of scanning electron microscopy (Figure 12) further confirmed the correlation between temperature changes and the nanostructure. The DEG-modified Ca(OH)_2_ presented an ultrathin nanosheet structure with abundant porous channels, which not only contributed to the high SSA (43.24 m^2^/g) but also increased the exposure of surface hydroxyl groups. Compared with the dense micro-sized particles of pure water-hydrated Ca(OH)_2_, the loose nanostructure of DEG-modified samples accelerated the mass transfer during decomposition and reduced the energy barrier for -OH removal, thus leading to the observed temperature shift [29].

Third, the high surface reactivity is evidenced by minor residual carbonation. A slight endotherm accompanied by a small mass loss is observed at 690.7 °C. While such peaks were formerly attributed to lattice relaxation, this feature is characteristic of the decomposition of trace calcium carbonate (CaCO_3_) [33]. The extreme sensitivity of the synthesized Ca(OH)_2_ to atmospheric CO_2_—a result of the high SSA confirmed by BET analysis—results in rapid superficial carbonation during sample handling. This atmospheric instability, paradoxically, demonstrates the superior chemical reactivity of the synthesized material compared to inert commercial lime.

### 3.3. Mechanistic Analysis

#### 3.3.1. Defect Engineering and Catalytic Decomposition of Limestone

The improvement of CaO reactivity via salt pretreatment is fundamentally governed by lattice defect engineering and the Kirkendall effect, rather than a mere cleaning of the particle surface.

Lattice Strain and Activation Energy: As evidenced by the Williamson–Hall analysis (Table 2), the incorporation of K^+^ ions (radius 1.38 Å) into the interstices of the smaller Ca^2+^ lattice (1.00 Å) induces substantial microstrain. This structural distortion destabilizes the thermodynamic equilibrium of the CaCO_3_ crystal lattice. According to the Arrhenius equation, this high-energy initial state reduces the activation energy (Ea) of the rate-determining step in the decomposition process, specifically the cleavage of the Ca-O bond. This is reflected in the experimentally observed 42.6 °C reduction in decomposition temperature. Simultaneously, the potassium salt acts as a mineralizer, forming a transient liquid phase at grain boundaries. This liquid phase facilitates the outward diffusion of CO_2_ and significantly lowers the energy barrier for CaCO_3_ dissociation.

Chemical Etching and Pore Development: Concurrently, NO_3_^−^ ions function as chemical etchants at elevated temperatures. They preferentially attack high-energy grain boundaries, creating pores that facilitate the rapid outward diffusion of CO_2_. This mechanism prevents the “pore blockade” phenomenon frequently observed during traditional calcination, leading to the development of a highly porous and interconnected CaO microstructure, which is optimal for subsequent hydration.

#### 3.3.2. Crystal Habit Modification via DEG-Mediated Hydration

The specific stereochemical interaction between diethylene glycol (DEG) and emerging portlandite crystals is the fundamental basis for the formation of high-SSA Ca(OH)_2_ nanosheets.

Selective Adsorption and Growth Inhibition: DEG functions as a crystal habit modifier in aqueous media. The etheric oxygen atoms and hydroxyl groups within the DEG molecule possess high electron density, enabling them to chelate Ca^2+^ ions on the crystal surface through hydrogen bonding and coordination. The surface energy of the Ca(OH)_2_ (001) basal plane is theoretically lower than that of the (100) or (010) prism planes. DEG molecules selectively bind to these (001) faces, forming a protective molecular shield.

Mechanism of Steric Hindrance: This adsorbed organic layer creates significant steric hindrance, effectively poisoning crystal growth along the c-axis (the stacking direction). Consequently, crystal growth is kinetically driven along the a-axes and b-axes. This process transforms the thermodynamically stable, thick hexagonal prisms into kinetically controlled, ultrathin nanosheets.

## 4. Conclusions

This study successfully established an integrated “Defect Engineering-Habit Modification” protocol for synthesizing high-reactivity Ca(OH)_2_ nanosheets from bulk limestone. The primary scientific findings are summarized as follows:

Thermodynamic Activation of Precursors: The KNO_3_-based pre-immersion protocol transcends simple surface cleaning; it introduces K^+^ interstitial defects that induce significant lattice strain (increasing microstrain to 1.24 × 10^−3^) and facilitate a flux-assisted decomposition pathway. This mechanism thermodynamically destabilizes the CaCO_3_ lattice, effectively lowering the decomposition temperature by approximately 42 °C. This results in a CaO precursor with enhanced porosity and intrinsic reactivity.

Stereochemical Control of Crystal Growth: High-SSA Ca(OH)_2_ is formed via a DEG-mediated anisotropic growth mechanism. DEG functions as a facet-selective capping agent, preferentially adsorbing onto the (001) basal planes of Ca(OH)_2_ and inhibiting c-axis stacking through steric hindrance. This redirective growth forces the crystallization of ultrathin, crumpled nanosheets instead of conventional bulk platelets.

Optimal Synthesis Conditions: The critical balance between growth inhibition and mass transport occurs at a DEG concentration of 70 wt%. Under these conditions, the system achieves its maximum specific surface area of 43.24 m^2^/g (a 142% increase relative to conventional hydration) and a high conversion efficiency of 98.5%. Deviating from this optimum leads to either poor morphological control (<50 wt%) or viscosity-induced agglomeration (>80 wt%).

Industrial Significance: The synthesized material—characterized by high purity, the absence of organic residues, and superior surface activity—demonstrates significant potential for applications in flue gas desulfurization and thermochemical energy storage. This protocol offers a robust pathway to bypass the efficiency bottlenecks associated with commercial lime sorbents.

## Figures and Tables

**Table 1:** Chemical Composition of Limestone (wt%).

CaO	SiO_2_	MgO	Loss
53.77	1.84	0.73	43.05

**Table 2:** Structural parameters of the prepared active CaO samples derived from Williamson–Hall analysis.

Sample	2*θ*_(200)_ (deg)	FWHM (°)	Lattice Parameter α (Å)	Crystallite Size (nm)	Microstrain *ε* (×10^−3^)
A0	37.55	0.195	4.787	64.6	0.93
A1	37.40	0.171	4.805	121.2	1.24
A2	37.45	0.184	4.799	78.4	1.00
A3	37.45	0.179	4.799	98.1	1.19
A4	37.47	0.170	4.797	90.0	1.11

**Table 3:** Calcium Oxide Sample Activity Test.

	1	2	3	Average Value	Standard Deviation
A0	348.9 mL	364.5 mL	357.9 mL	357.1 mL	7.88 mL
A1	430.2 mL	440.5 mL	436.4 mL	435.7 mL	5.19 mL
A2	397.2 mL	402.5 mL	405.7 mL	401.8 mL	4.58 mL
A3	410.6 mL	419.6 mL	416.6 mL	415.6 mL	4.58 mL
A4	421.1 mL	431.0 mL	426.5 mL	426.2 mL	4.96 mL

**Table 4:** Proportional Composition of Solvent DEG and Deionized Water.

Sample	DEG/wt%	Deionized Water/wt%
D1	50	50
D2	60	40
D3	70	30
D4	80	20
D5	90	10

**Table 5:** Quantitative Phase Analysis of Ca(OH)_2_ Synthesized with Varying DEG Content.

Sample	Ca(OH)_2_/%	CaO/%	CaCO_3_/%
50 wt% DEG + 50 wt% H_2_O	93.3	5.7	1.0
60 wt% DEG + 40 wt% H_2_O	95.8	2.9	1.2
70 wt% DEG + 30 wt% H_2_O	98.5	0	1.5
80 wt% DEG + 20 wt% H_2_O	92.4	6.2	1.4
90 wt% DEG + 10 wt% H_2_O	92.1	6.6	1.3
100 wt% H_2_O	90.5	8.4	1.1

## Data Availability

The original contributions presented in this study are included in the article. Further inquiries can be directed to the corresponding author.

## Appendix A. Comparison of BET-Specific Surface Area of Ca(OH)_2_ Prepared by Different Methods

**Synthesis Method**	**BET SSA (m^2^/g)**	**Reference**	**Advantages and Disadvantages**
Conventional water-only digestion	13–18 m^2^/g	[24]	Weak dissipation capacity and narrow application scope
The surfactant water uniform mixing method	24.67–47.65 m^2^/g	[34]	Simple process, the raw material cost is low and easily obtainable, and it is suitable for large-scale industrial applications
Homogeneous precipitation method	Up to 96 m^2^/g	[35]	High purity, uniform particles, but high cost, long reaction period, and high energy consumption
PEG-assisted digestion	40–48 m^2^/g	[36]	Good dispersion, high dissolution efficiency, and the introduction of organic background
This work	43.24 m^2^/g	This work	Good emulsifying and dispersing effects, high activity, high specific surface area

## References

[1] Ponomar V., Yliniemi J., Kilpimaa K. (2024). Influence of calcium oxide and hydroxide on non-ferrous metallurgical slag hydration in alkaline environments. Cem. Concr. Compos..

[2] Török P., Halasiné-Varga I., Duvivier L., Maimi M., Hubert O., Minnaar S., Sipos P., du Plessis C., Kutus B. (2025). Lithium Carbonate Conversion to Lithium Hydroxide Using Calcium Hydroxide: Equilibrium is Governed by Vaterite Formation. Inorg. Chem..

[3] Zhao L., Wang C., Na S., Jin Y., Kang W., Zhu J., Zhang W., Bian Y., Shah S.P. (2025). A study of blast furnace slag on the mechanical properties improvement and microstructure of hemihydrate phosphogypsum pretreated by calcium hydroxide. Case Stud. Constr. Mater..

[4] Liu M., Ding W., Chang J., Wan J., Liu W., Duan Y. (2025). Pilot study on coke oven flue gas injection desulfurization using highly active modified calcium hydroxide desulfurizer. Asia-Pac. J. Chem. Eng..

[5] Ma X., Wu H., Chang L., Wang J., Wang Y., Cui S. (2022). The influence of calcium hydroxide crystal morphology on the desulfurization of cement kiln flue gas. J. Mater. Sci..

[6] Costagliola M.A., Prati M.V., Perretta G. (2022). Post combustion CO_2_ capture with calcium and lithium hydroxide. Sci. Rep..

[7] Funayama S., Takasu H., Kim S.T., Kato Y. (2020). Thermochemical storage performance of a packed bed of calcium hydroxide composite with a silicon-based ceramic honeycomb support. Energy.

[8] Huang C., Xu M., Huai X. (2019). Experimental investigation on thermodynamic and kinetic of calcium hydroxide dehydration with hexagonal boron nitride doping for thermochemical energy storage. Chem. Eng. Sci..

[9] Chepkonga B.J., Koech L., Rutto H.L. (2023). The performance of hydrated lime derived from industrial brine sludge waste in spray dry scrubbing of SO_2_. Eng. Proc..

[10] Xu M., Huai X., Cai J. (2017). Agglomeration behavior of calcium hydroxide/calcium oxide as thermochemical heat storage material: A reactive molecular dynamics study. J. Phys. Chem. C.

[11] Chen J., Li L., Liu L., Zhang D., Jiang C., Zhu H., Liao Q., Wang F. (2025). Tailored Synthesis of Ca(OH)_2_ with High Specific Surface Area from Quicklime for Removal of Cd(II) from Solution. ChemistrySelect.

[12] Moropoulou A., Bakolas A., Aggelakopoulou E. (2001). The effects of limestone characteristics and calcination temperature to the reactivity of the quicklime. Cem. Concr. Res..

[13] Takkinen S., Saastamoinen J., Hyppänen T. (2012). Heat and mass transfer in calcination of limestone particles. AIChE J..

[14] Duan S., Li B., Rong W. (2022). Numerical simulation study of gas-solid heat transfer and decomposition processes of limestone calcined with blast furnace gas in a parallel flow regenerative lime kiln. Materials.

[15] Sun S., Chen Z., Xu Y., Wang Y., Zhang Y., Dejoie C., Xu S., Xu X., Wu C. (2023). Potassium-promoted limestone for preferential direct hydrogenation of carbonates in integrated CO_2_ capture and utilization. JACS Au.

[16] Liendo F., Arduino M., Deorsola F.A., Bensaid S. (2022). Nucleation and growth kinetics of CaCO_3_ crystals in the presence of foreign monovalent ions. J. Cryst. Growth.

[17] Arcenegui-Troya J., Sánchez-Jiménez P.E., Perejon A., Moreno V., Valverde J.M., Pérez-Maqueda L.A. (2021). Kinetics and cyclability of limestone (CaCO_3_) in presence of steam during calcination in the CaL scheme for thermochemical energy storage. Chem. Eng. J..

[18] Eriksson M., Sandström K., Carlborg M., Broström M. (2024). Impact of limestone surface impurities on quicklime product quality. Minerals.

[19] Pan Z., Jian C., Peng Z., Fu X., He R., Yue T., Sun W. (2024). Study on Process Mineralogy of the Combined Copper Oxide Ore in Tibet and Acid Leaching Behavior with Calcium Fluoride. Minerals.

[20] Liu W., An H., Qin C., Yin J., Wang G., Feng B., Xu M. (2012). Performance enhancement of calcium oxide sorbents for cyclic CO_2_ capture—A review. Energy Fuels.

[21] Karunadasa K.S., Manoratne C.H., Pitawala H.M.T.G.A., Rajapakse R. (2019). Thermal decomposition of calcium carbonate (calcite polymorph) as examined by in-situ high-temperature X-ray powder diffraction. J. Phys. Chem. Solids.

[22] Wang J., Yin J., Kong X. (2022). Influences of PCE superplasticizers with varied architectures on the formation and morphology of calcium hydroxide crystals. Cem. Concr. Res..

[23] Martínez-Arredondo A., García-Vera V.E., Navarro-Moreno D., Tenza-Abril A.J., Lanzón M. (2024). Calcium hydroxide, magnesium hydroxide, silicon dioxide nanoparticles and their combinations as consolidants for lime mortars and gypsum plasters. J. Cult. Herit..

[24] Yan D., He R., Wu X., Zhu Y. (2025). An investigation of the surface structure of surfactants modified calcium hydroxide and enhancement of dry flue gas desulfurization performance. Res. Chem. Intermed..

[25] Sun R., Shen H., Lv X., Wang Y., Hu T. (2024). Solution combustion synthesis of MgO-stabilized CaO sorbents using polyethylene glycol as fuel and dispersant. RSC Adv..

[26] Rodriguez-Navarro C., Burgos-Cara A., Lorenzo F.D., Ruiz-Agudo E., Elert K. (2020). Nonclassical crystallization of calcium hydroxide via amorphous precursors and the role of additives. Cryst. Growth Des..

[27] Wang J., Kong X., Yin J. (2020). Impacts of two alkanolamines on crystallization and morphology of calcium hydroxide. Cem. Concr. Res..

[28] Fan B., Cheng F., Cheng H. (2021). Research progress of calcium-based adsorbents for CO_2_ capture and anti-sintering modification. J. Fuel Chem. Technol..

[29] Yan D., Zhu Y., Zhao J., Zhang Q., Wang Y., Yang S. (2024). Synthesis and utilization of polyol-modified high specific surface area Ca(OH)_2_: An investigation. Environ. Sci. Pollut. Res..

[30] Dai W., Gong C., Lu L., Cheng X. (2018). Effect of MgO on calcination and properties of belite-barium calcium sulphoaluminate cement clinker with Na_2_O and K_2_O. Ceram. Silikáty.

[31] Takaki S., Masumura T., Tsuchiyama T. (2018). Proposal of simplified modified Williamson-Hall equation. ISIJ Int..

[32] Abdelmoaty A., Mousa S. (2024). Synthesis and characterization of hydroxyapatite nanoparticles from calcium hydroxide fouled with gases evolved from smokestack of glass industry. Sci. Rep..

[33] Zheng J., Huang J., Tao L., Li Z., Wang Q. (2020). A multifaceted kinetic model for the thermal decomposition of calcium carbonate. Crystals.

[34] Shin H.G., Kim H., Kim Y.N., Lee H.S. (2009). Preparation and characterization of high surface area calcium hydroxide sorbent for SO_2_ removal. Curr. Appl. Phys..

[35] Daniele V., Taglieri G. (2012). Synthesis of Ca(OH)_2_ nanoparticles with the addition of Triton X-100. Protective treatments on natural stones: Preliminary results. J. Cult. Herit..

[36] Xing G., Li S., Wang Y., Zhang Y., Wang W., Wang W., Qi L. (2025). From laboratory to industry: Preparation and mechanism analysis of organic additive-modified high-activity calcium hydroxide desulfurizer in low-temperature dry desulfurization. Sep. Purif. Technol..

